# Red Cabbage Juice-Mediated Gut Microbiota Modulation Improves Intestinal Epithelial Homeostasis and Ameliorates Colitis

**DOI:** 10.3390/ijms25010539

**Published:** 2023-12-30

**Authors:** Emily Jean Wilson, Nagabhishek Sirpu Natesh, Parsa Ghadermazi, Ramesh Pothuraju, Dipakkumar R. Prajapati, Sanjit Pandey, Jussuf T. Kaifi, John R. Dodam, Jeffrey N. Bryan, Christian L. Lorson, Aude A. Watrelot, Jason M. Foster, Thomas J. Mansell, Siu Hung Joshua Chan, Surinder K. Batra, Jeyamkondan Subbiah, Satyanarayana Rachagani

**Affiliations:** 1 Department of Biological Systems Engineering, University of Nebraska-Lincoln, Lincoln, NE 68583, USA; wilson.emilyjean@gmail.com; 2 Department of Veterinary Medicine and Surgery, University of Missouri, Columbia, MO 65201, USA; snagabhishek@missouri.edu (N.S.N.); dodamj@missouri.edu (J.R.D.); bryanjn@missouri.edu (J.N.B.); 3 Roy Blunt NextGen Precision Health Institute, University of Missouri, Columbia, MO 65211, USA; 4 Department of Chemical and Biological Engineering, Colorado State University, Fort Collins, CO 80523, USA; parsa.ghadermazi@colostate.edu (P.G.);; 5 Department of Biochemistry and Molecular Biology, University of Nebraska Medical Center, Omaha, NE 68198, USA; 6 Department of Pathology and Microbiology, University of Nebraska Medical Center, Omaha, NE 68198, USA; dipakdoctor@gmail.com; 7 Department of Genetics, Cell Biology and Anatomy, University of Nebraska Medical Center, Omaha, NE 68198, USA; sanjit.pandey@unmc.edu; 8 Department of Surgery, School of Medicine, University of Missouri, Columbia, MO 65211, USA; kaifij@health.missouri.edu; 9 Department of Veterinary Pathobiology, University of Missouri, Columbia, MO 65211, USA; lorsonc@missouri.edu; 10 Department of Food Science and Human Nutrition, Iowa State University, Ames, IA 50011, USA; watrelot@iastate.edu; 11 Department of Surgery, Division of Surgical Oncology, University of Nebraska Medical Center, Omaha, NE 68198, USA; 12 Department of Chemical and Biological Engineering, Iowa State University, Ames, IA 50011, USA; mansell@iastate.edu; 13 Department of Food Science, University of Arkansas, Fayetteville, AR 72701, USA; jsubbiah@uark.edu

**Keywords:** red cabbage, Dextran Sodium Sulfate (DSS), colitis, gut microbiota, inflammatory bowel disease (IBD), short-chain fatty acids (SCFAs)

## Abstract

Gut microbiota plays a crucial role in inflammatory bowel diseases (IBD) and can potentially prevent IBD through microbial-derived metabolites, making it a promising therapeutic avenue. Recent evidence suggests that despite an unclear underlying mechanism, red cabbage juice (RCJ) alleviates Dextran Sodium Sulfate (DSS)-induced colitis in mice. Thus, the study aims to unravel the molecular mechanism by which RCJ modulates the gut microbiota to alleviate DSS-induced colitis in mice. Using C57BL/6J mice, we evaluated RCJ’s protective role in DSS-induced colitis through two cycles of 3% DSS. Mice were daily gavaged with PBS or RCJ until the endpoint, and gut microbiota composition was analyzed via shotgun metagenomics. RCJ treatment significantly improved body weight (*p* ≤ 0.001), survival in mice (*p* < 0.001) and reduced disease activity index (DAI) scores. Further, RCJ improved colonic barrier integrity by enhancing the expression of protective colonic mucins (*p* < 0.001) and tight junction proteins (*p* ≤ 0.01) in RCJ + DSS-treated mice compared to the DSS group. Shotgun metagenomic analysis revealed an enrichment of short-chain fatty acids (SCFAs)-producing bacteria (*p* < 0.05), leading to increased Peroxisome Proliferator-Activated Receptor Gamma (PPAR-γ) activation (*p* ≤ 0.001). This, in turn, resulted in repression of the nuclear factor κB (NFκB) signaling pathway, causing decreased production of inflammatory cytokines and chemokines. Our study demonstrates colitis remission in a DSS-induced mouse model, showcasing RCJ as a potential modulator for gut microbiota and metabolites, with promising implications for IBD prevention and treatment.

## 1. Introduction

Inflammatory bowel diseases (IBDs), including Crohn’s disease (CD) and ulcerative colitis (UC), are a significant health problem around the world [1,2]. In 2015, the Centers for Disease Control and Prevention (CDC) estimated that 1.3% of all adults, accounting for a total of 3.1 million patients in the USA and 1 in 123 individuals in the UK, are suffering from IBD. At least 10 million IBD cases have been reported worldwide [1,3].

The primary clinical symptoms of IBD include acute abdominal pain, rectal bleeding, weight loss, anemia, hematochezia, diarrhea, negative effects on the immune system, and mortality risk [4]. Diagnosis and determination of IBD type and severity depend on clinical evaluation, tests, imaging, and endoscopic procedures [5,6]. Distinguishing between CD and UC entails recognizing that CD may exhibit cobblestoning throughout the GI tract, while UC generally manifests in the rectum but may extend proximally in a continuous fashion. Colonoscopy and capsule endoscopy play pivotal roles in diagnosing and distinguishing between CD and UC [7]. Although the complete pathogenesis of IBD is yet to be fully understood, impairment of the epithelial cellular barrier, inflammation-mediated immune dysfunction, and dysbiosis of the gut microbiota are hallmarks of IBD [8,9]. Typically, intestinal epithelial barrier disruption leads to the translocation of microbiota from the lumen to the lamina propria, eventually triggering inflammation [10].

The current treatments for IBD include various pharmacological interventions. The primary treatment for IBD is monoclonal antibodies. However, most patients lose responsiveness with time [11]. Anti-TNF agents, such as infliximab, immunosuppressives such as azathioprine and α4β7 integrin blocker, vedolizumab, have been introduced for IBD therapy and have helped to reduce corticosteroid use [7,12]. Additionally, the long-term use of antibiotics, including ciprofloxacin and metronidazole, is widespread in patients with IBD, leading to antibiotic resistance and negatively altering the gut microbiota [13]. Regardless of various IBD treatment options, the outcome is associated with significant risks and limitations, mainly due to limited effectiveness of current drugs and high clinical heterogeneity of patient presentation. Thus, there is an urgent need for alternative and effective treatments to combat the limited effectiveness of current drugs and the recurring cases of IBD [7]. 

Recent in vitro and in vivo studies have highlighted that gut dysbiosis may play a vital role in IBD pathogenesis [14,15,16,17]. The role of gut dysbiosis is being studied with nutraceuticals, including prebiotics, probiotics, symbiotics and attractive therapies like fecal transplantation for alleviating intestinal inflammation. Gut microbiota plays a significant role in maintaining the gastrointestinal (GI) tracts’ homeostasis. It also acts as a metabolic organ and contributes to health by performing various physiological functions. Deviation in the gut flora composition is involved in multiple disease pathologies, including IBD [16]. Therefore, incorporating prebiotics and probiotics, extending beyond dietary choices, emerges as a powerful over-the-counter tool for preserving and maintaining healthy gut microbiota [18]. Thus, immediate basic science research efforts are focused on alternative strategies like nutraceuticals to modulate gut microbiota to prevent the progression of IBD [19].

Nutraceuticals are pharmaceutical alternatives/food supplements with physiological benefits. Nutraceuticals can treat IBD directly, as they contain bioactive compounds from plants, such as polyphenols and poly/oligosaccharides (carbohydrates that are indigestible by host digestive enzymes) that benefit the intestinal epithelium. Polyphenols (anthocyanins) have been shown to have direct anti-inflammatory and antioxidant effects on the intestinal epithelium, thus acting as therapeutics for IBD patients [20]. Moreover, nutraceuticals can also treat IBD by modulating the gut microbiota [19] and their derived metabolites. For example, polyphenol compounds present in nutraceuticals potentially enrich *Bifidobacteria* and *Lactobacilli* sp. to thrive in the gut; in turn, these bacteria release secondary metabolites such as SCFAs with anti-inflammatory properties, acting as potential therapeutics for IBD patients [21].

Between SCFAs derived from the gut microbiota and subsequent inflammatory changes in IBD through clinical studies. The SCFAs produced from gut microbiota, facilitated by the fermentation of dietary fiber, seem beneficial in preserving gut microbiota-derived SCFAs and modulating secondary changes to regulate immune responses. This offers a potential therapeutic avenue for IBD. In vitro studies unveil SCFAs’ impact on gut epithelial barrier function and immune regulation, thereby promoting mucosal healing by suppressing inflammation. There is a correlation between reduced fecal SCFAs levels and active IBD, emphasizing SCFAs’ role in disease activity. Insights from various studies illuminated the intricate relationship between SCFAs, gut microbiota, and inflammatory responses in IBD [22,23,24,25].

Red cabbage (*Brassica oleracea* L. *var. capitata f. rubra*) juice extract is widely used in traditional medicine. It contains rich minerals, oligosaccharides, and several bioactive substances such as glucosinolates (GSLs), indole-sulfur phytoalexins, S-methylmethionine, and phenolic compounds such as flavonol glycosides, acylated anthocyanins, and hydroxycinnamic acid derivatives [26,27]. Among them, anthocyanins, major polyphenol pigments, have been reported to reduce acute and chronic colitis in mouse models [28]. GSLs, hydrolyzed by myrosinase, produce bioactive compounds, such as indoles and isothiocyanates (ITCs) [29]. In a recent in vitro study, *Brassica* species phytochemicals showed decreased cytokine IL6 and increased IL10 expression. Additionally, phytochemicals like carotenoids and polyphenols exhibited a reduction in protein expression of catalase, glutathione transferase, and superoxide dismutase (SOD) [30]. The increased consumption of *Brassica* vegetables was linked to a reduced relative abundance of sulphate-reducing bacteria (SRB), leading to beneficial gastrointestinal health [31]. 

Thus, our current study aimed to explore the nutraceutical potential of RCJ. We investigated whether RCJ could ameliorate IBD through modulation of the gut microbiota in a DSS-induced colitis mouse model. 

## 2. Results

### 2.1. Bioactive Compounds Remain Active despite Freezing and PEF Treatment

To understand whether PEF treatment, freezing, and thawing would significantly affect its biological activity, different biological parameters were tested. There was no substantial change in the bioactivity of RCJ (Supplementary File S1 Table S1). Furthermore, there was no significant change in total phenolic concentrationand free anthocyanin content in RCJ (Supplementary File S1 Table S1).

Chemical parameter analysis was then carried out. Four red cabbages were procured (total weight, 3.8 kg), and 1.4 liters of juice was obtained using a food processor and filtered with a cheesecloth. The pH of RCJ was 6.42 ± 0.05 with dissolved solids of approximately 6.4 ± 0.2 gms. RCJ juice contained 18.5 ± 0.2 g (gms) of glucose, 15.1 ± 0.2 gms of fructose, 0.8 ± 0.1 gms of citric acid, 3 ± 0.3 gms of malic acid and 34.2 ± 2.1 gms of unknown acids per liter of RCJ (Supplementary File S1 Table S1).

Spectrophotometry analysis of RCJ revealed that 382.5 ± 93.5 mg of total phenolic compound and 257.7 ± 3.1 mg of anthocyanins are present in 1 L of RCJ (Supplementary File S1 Table S1). Next, high-performance liquid chromatography with diode-array detection (HPLC-DAD) was performed to determine the composition of polyphenols in RCJ. Approximately eight monomeric polyphenols were identified in RCJ (Table S2). In addition to polyphenols, RCJ contains 254 ± 23.6 mg and 55 ± 2.3 mg of free anthocyanins, and total hydroxycinnamic acid per liter of RCJ. After the characterization of organic acids and sugars in RCJ, it was found that RCJ had a high concentration of organic acid compounds. Thus, RCJ was further analyzed for glycosyl composition (sugar residue analysis). This will quantify the monosaccharide composition of polysaccharides, oligosaccharides, or glycoproteins present in RCJ by gas chromatography–mass spectrometry (GC-MS) using TMS-derivatized monosaccharides. Our results revealed that the carbohydrates in RCJ are mainly composed of glucose residues, with galactose, fructose, arabinose, and mannose as the minor sugar components (Supplementary File S1 Figure S1). Detailed calculations of the molecular percentage of monosaccharides and the total carbohydrate percentage by weight for the RCJ sample are shown in (Supplementary File S1 Table S3).

### 2.2. Prophylactic RCJ Intervention Alleviates DSS-Induced Colitis in Mice

**Figure 1:**
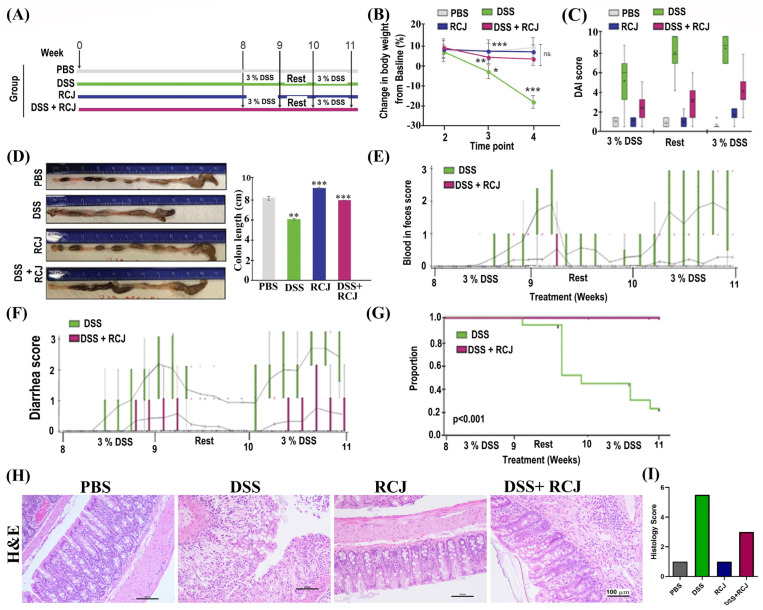
(**A**) Schematic diagram of the in vivo experimental design and access to water and standard feed. (**B**) Effect of DSS and RCJ on body weight where time point 1 is the baseline before treatments began. Time point 2 is during the RCJ treatments. Time point 3 is after the DSS administration began. Time point 4 is after a rest period. Error bars are the confidence interval (95%). Data were presented as mean ± SEM (*n* = 15 per group). (**C**) kinetics of daily disease activity index (DAI) scores throughout the study duration (**D**). Effect of RCJ and DSS on colon length. Effect of RCJ and DSS on (**E**) Blood in feces scores. (**F**) Diarrhea scores. (**G**) Survival curve. (**H**) H&E–stained colon section. (**I**) Histological scores of the colon (*n* = 15 per group). Scale bars represent 100 µm. Statistical significance was determined using one-way ANOVA, followed by the Tukey test. ns = non-significant; * *p* ≤ 0.05, ** *p* ≤ 0.01, *** *p* ≤ 0.001.

To directly assess the effect of RCJ on DSS-induced colitis development, C57BL/6 mice were divided into four groups: PBS, DSS, RCJ, and DSS + RCJ (Figure 1A). The DSS group showed a significant decrease in body weight (Figure 1B), colon length (Figure 1D) and higher blood and diarrhea scores (Figure 1E,F), whereas RCJ administration reverted to normal. Furthermore, compared with the DSS group, RCJ supplementation resulted in a lower disease activity index (DAI) (Figure 1C).

Subsequently, the survival benefits were analyzed using RCJ in combination with DSS. In mice treated with DSS, the Kaplan-Meier Survival curve revealed a higher percentage of deaths due to severe colitis than in the other groups (*p* < 0.001). (Figure 1G). No deaths were observed in the PBS, RCJ, or RCJ + DSS groups. Times were censored at the end of the study owing to deaths unrelated to colitis, such as misadministration of oral gavage. 

In addition, H&E staining showed increased crypt depth, submucosal edema, macroscopic spaces between crypts, less hyperplasia of crypts, low epithelial cell damage, damaged brush borders (villi), reduced mononuclear cell infiltration in the submucosa, and reduced colon inflammation upon RCJ supplementation (Figure 1H). Moreover, an increase in the overall histological score (parameters for the histology score were architectural damage, extent of inflammation, presence of chronic inflammatory infiltrate) was observed in the RCJ group compared to the DSS alone group (Figure 1I).

### 2.3. RCJ Ameliorates Colitis by Regulating Intestinal Barrier Function and DSS-Induced Oxidative Stress in the Intestinal Mucosa

**Figure 2:**
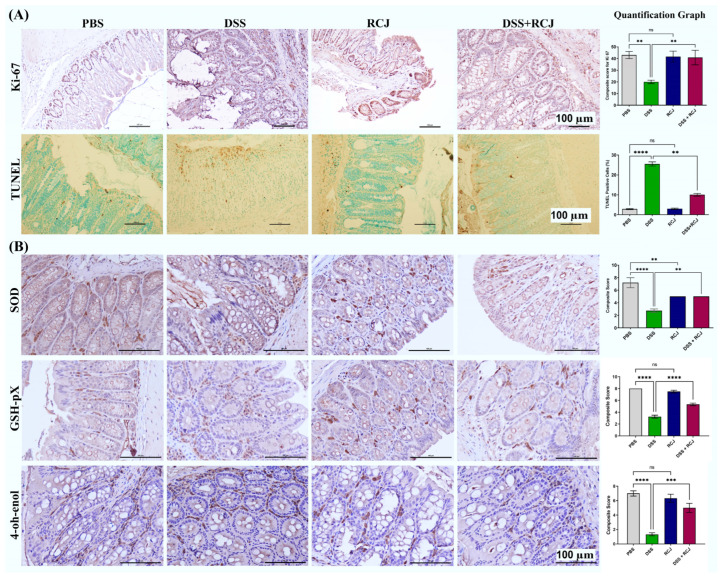
RCJ attenuated oxidative stress and colonic damage. (**A**) IHC staining for Ki67 in colonic epithelium to assess epithelial cell proliferation and TUNEL-positive nuclei (apoptotic cells) in the colonic epithelium in brown (**B**) Representative colons IHC for oxidative stress marker from each treatment group;. Error bars in the histograms are the standard error of the mean. Scale bars represent 100 µm. Statistical significance was determined using one-way ANOVA, followed by the Tukey test. ns = non-significant; ** *p* ≤ 0.01, *** *p* ≤ 0.001, **** *p* ≤ 0.0001.

Healthy epithelial cells are critical for maintaining intestinal barrier function. Proliferation and apoptosis are two key factors in the differentiation of healthy epithelial cells. Colonic epithelial cell proliferation was assessed by Ki-67 staining. The DSS + RCJ group showed a significantly higher number (*p* < 0.05) of Ki-67 positive cells when compared to the DSS group (Figure 2A). Furthermore, RCJ treatment reduced the number of TUNEL-positive nuclei in the colonic epithelium compared to that in the DSS group (Figure 2A). The DSS + RCJ group showed significantly reduced TUNEL-positive nuclei, indicating reduced apoptosis. Next, the influence of RCJ on oxidative stress, superoxide dismutase (SOD), 4-Hydroxynonenal (4-OH-enol), and glutathione peroxidase 4 (GPX4) in the colon tissue was measured. Compared with the DSS group, the DSS + RCJ group showed suppressed concentrations of SOD, GPX4, and 4-OH-enol. Compared with the PBS controls, initiation of colitis was attenuated mainly by RCJ (Figure 2B). These results show that RCJ improved the integrity of the intestinal epithelial barrier while restoring the intestinal barrier function. Together, these results showed that RCJ treatment markedly ameliorated DSS-induced colitis.

**Figure 3:**
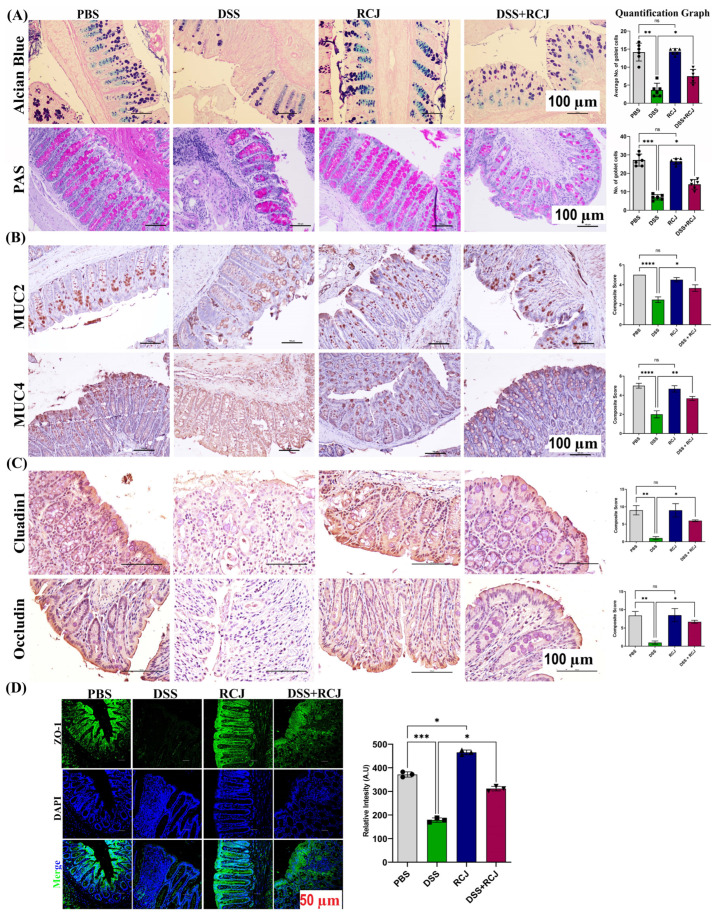
RCJ ameliorates colitis by regulating the intestinal barrier function. (**A**) Alcian blue staining denoting the mucin-secreting goblet cells in the colonic epithelia and PAS-positive cells denoting acid and neutral mucin. (**B**) IHC staining for MUC2 and MUC4, stained to understand the protective mucin layer. (**C**) IHC staining for tight junction markers claudin and occludin. (**D**) Immunostaining for colonic ZO-1, (green) counterstained with the nuclear stain DAPI (blue) and elative intensity, quantitative analysis of the fluorescence by Image J (Fiji 1.54F). All values represent the means ± SD; error bars in the histograms are the standard error of the mean. Scale bars represent 100 µm for the IHC and 50 µm for IF. Statistical significance was determined using one-way ANOVA, followed by the Tukey test. ns: non-significant * *p* ≤ 0.05, ** *p* ≤ 0.01, *** *p* ≤ 0.001, **** *p* ≤ 0.0001.

H&E staining showed that the DSS group had intense, severe epithelial cell damage, submucosal edema, including shortening and hyperplasia of crypts, inflammatory lesions, macroscopic spaces between crypts, and severe inflammatory cellular infiltrate in the submucosa. RCJ treatment notably ameliorated the colonic inflammatory symptoms, including reduced inflammatory cell infiltrate, relative submucosal edema, intact surface epithelium, normal crypt glands, mild submucosal edema, and the condition of the colon was close to that of PBS mice. The DSS-treated group showed a significant reduction in the thickness of the colonic epithelial mucosa, which was attenuated to near normal levels. Thus, to assess the effect of RCJ on the colonic mucosal barrier, Alcian blue and PAS staining were used to check mucin-secreting goblet cells in the colonic epithelia (Figure 3A). Next, intestinal homeostatic mucins (MUC2 and MUC4) and supplementation with RCJ resulted in a significant (*p* < 0.001) increase in the expression of colonic mucins (Figure 3B). Furthermore, RCJ-treated animals showed improved intestinal barrier function and increased expression of tight junction proteins, such as ZO-1, Occludin, and Claudin-1 (Figure 3C,D).

### 2.4. Prophylactic RCJ Intervention Alleviated Colonic Pro-Inflammatory Status

**Figure 4:**
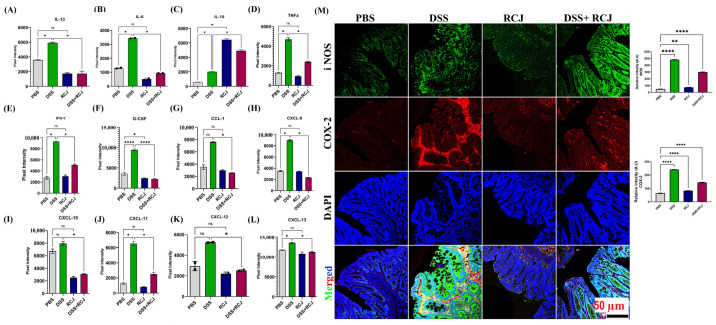
Effect of RCJ on colonic proinflammatory status (**A**–**L**). Graphs show a quantified expression of pro- and anti-inflammatory cytokines and chemokines using a cytokine array. (**M**) Immunofluorescence staining for iNOS (green), COX-2 (red) and counterstained with the nuclear stain DAPI (blue). Graphs show relative intensity, quantitative analysis of the fluorescence by Image J. All values represent the means ± SD; error bars in the histograms are the standard error of the mean. Scale bars represent 50 µm for IF. Statistical significance was determined using one-way ANOVA, followed by the Tukey test. ns: non-significant, * *p* ≤ 0.05, ** *p* ≤ 0.01, **** *p* ≤ 0.0001.

To elucidate how RCJ reduced colitis severity, the expression of pro- and anti-inflammatory cytokines and chemokines was quantified using a cytokine array (IL-1α, IL-1β, IL-3, IL-6, IL-10, IL-16, IL-17, IL-23, IL-27, CCL1, CXCL1, CXCL9, CXCL10, CXCL11, G-CSF, GM-CSF, TNF-α, and IFN-γ). DSS-treated mice had elevated levels of crucial pro-inflammatory cytokines such as TNF-α, IFN-γ, IL-1β, and IL-6, and chemokines such as CXCL1 and CXCL9–11. In contrast, RCJ treatment resulted in significantly (*p* > 0.005) lower levels of various pro-inflammatory cytokines and chemokines and elevated levels of the anti-inflammatory cytokine IL-10 (Figure 4A–L). Furthermore, iNOS and COX-2 were also reduced by RCJ treatment (*p* < 0.05 and *p* < 0.001, respectively) (Figure 4M). These results suggest that RCJ can diminish the pro-inflammatory cytokine response in DSS-induced colitis in mice.

### 2.5. Prophylactic RCJ Intervention Restores Microbiome Diversity and Content µ

**Figure 5:**
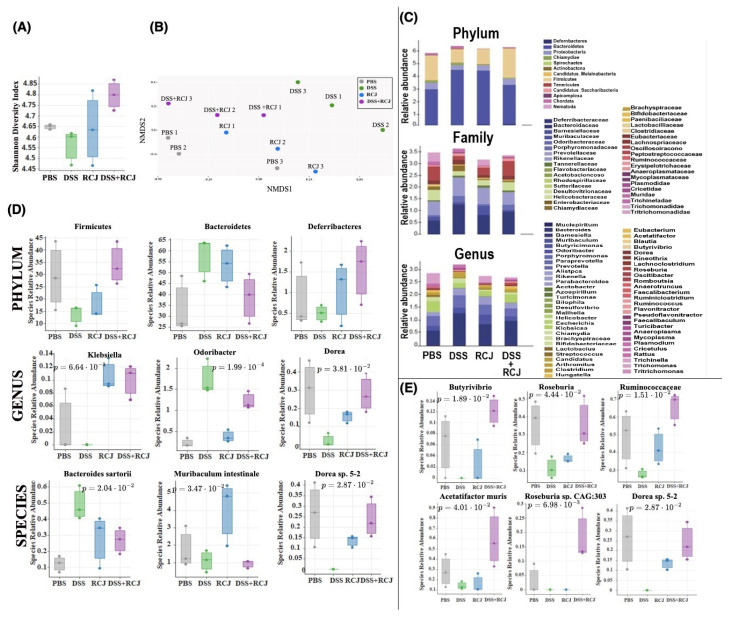
Effect of RCJ treatment on gut microbiota. (**A**) Alpha diversity using Shannon indices, a measure of evenness in a sample. (**B**) NMDS plots representing the samples’ closeness when compared to the control. (**C**) Relative abundance of taxa. (**D**) Relative abundance of significant organisms at phylum, genus, and species level. (**E**) Relative abundance of some reported SCFA–producing taxa. Statistical significance was determined using one-way ANOVA, followed by the Tukey test.

The release of inflammatory cytokines and chemokines, which could be due to an abnormal gut microbiota composition, would induce an altered immune response, including the release of inflammatory factors and aggregation of inflammatory cells. Our biochemical analysis showed that the presence of oligo- and polysaccharides in RCJ could modulate gut microbiota composition. To address this, shotgun metagenomic sequencing was performed to infer the taxonomy and functional changes in all the treatment groups. RCJ administration was explored on colon microbial alpha diversity using Shannon indices (a measure of evenness in the samples) on taxonomy analysis results at the species level. The DSS group showed a decrease in alpha diversity (Figure 5A). The existing carbohydrates/polysaccharides in RCJ could promote a higher alpha diversity.

Non-metric multi-dimensional scaling (NMDS) plots of taxonomy and pathway data consistently revealed that the microbiome in the RCJ + DSS group was more similar to the control samples than in the DSS group alone (Figure 5B). This supports the hypothesis that under DSS-induced colitis, RCJ partially restores the microbiome closer to a normal healthy microbiome.

### 2.6. RCJ Intervention Alters the SCFA-Producing Gut Microbiota Population

Impact of RCJ on cecal mucosa and luminal microbiota composition was studied using MEtaGenome ANalyzer (MEGAN) analysis. At the phylum level, we did not observe a compelling signal of differentially abundant phyla (Figure 5C), except for the following comparisons that were close to the significance threshold (Supplementary File S2). There is an increased abundance of *Bacteroidetes*, *Chlamydiae*, and *Chordata* and a decreased abundance of *Firmicutes* in a DSS-treated group of mice. However, when compared with PBS, RCJ, and RCJ + DSS groups, there is an increased abundance of *Firmicutes*, *Deferribacteres* and reduced abundance of *Bacteroidetes* and *Chordata* in the RCJ + DSS group (Figure 5D). 

A comprehensive set of significant taxa was provided in (Supplementary Files S1 and S2). Here, the focus was on the taxa that were found to be more relevant to colitis while being significant.

At the genus level, RCJ treatment significantly enriched *Muribaculum*, *Klebsiella*, and *Desulfovibrio* (Figure 5D and Supplementary File S1 Figure S4) (*p* < 0.05, 0.005, and 0.05, respectively) compared with the control group. *Ruminococcus* (Supplementary File S1 Figure S5A) and *Odoribacter* (Figure 5D) genera were significantly enriched in the DSS group compared to the control group (*p* < 0.05, 0.0413, and 0.0005, respectively). In contrast, *Lachnoclostridium* (Supplementary File S1 Figure S5A) and *Dorea* (Figure 5D) genera were significantly enriched in the RCJ + DSS group compared to the DSS-only group (*p*-values 0.05 and 0.05, respectively). Furthermore, *Bacteroides sartorii* (Figure 5D) and *Ruminococcus flavefacien* (Supplementary File S1 Figure S5A) species were enriched in the DSS group (*p* < 0.05 and 0.05, respectively). While *Clostridium* sp. CAG:557 (Supplementary File S1 Figure S5A) and *Dorea* sp. 5-2 (Figure 5D) were depleted in the DSS group (*p* < 0.005 and 0.05, respectively), *Muribaculum intestinale* (Figure 5D) was strongly enriched by RCJ treatment (*p* < 0.05). (Supplementary File S2) summarizes significant taxa at different taxonomic levels. 

Furthermore, LefSe analysis was performed to detect significantly different taxa at different taxonomic levels (Supplementary File S1 Figure S4). This allowed us to generate a cladogram representing the taxonomic relatedness of the significant taxa. The cladogram showed substantial differences in 106 taxa among the four treatment groups (PBS, RCJ, DSS, and DSS + RCJ) (Supplementary File S1 Figure S3). Red, green, blue, and purple indicate different groups, with the species classification at the phylum level, class, order, family, and genus shown from inside to outside. For instance, the cladogram clearly indicated that DSS treatment affected members of the *Bacteroidales* family. In contrast, the yellow nodes represent species with no significant differences. 

Furthermore, a few key taxa, such as *Butyrivibrio*, *Roseburia*, *Ruminococcaceae*, *Acetatifactor muris*, *Rosburia* Sp. CAG:303, *Dorea* Sp. 5-2, were found. They were more abundant in the DSS + RCJ group than in the DSS group (Figure 5E). These families of *Clostridia* have been reported to produce butyrate [32,33]. Additionally, these microbial taxa have been reported to be responsible for SCFA production [34,35,36]. 

**Figure 6:**
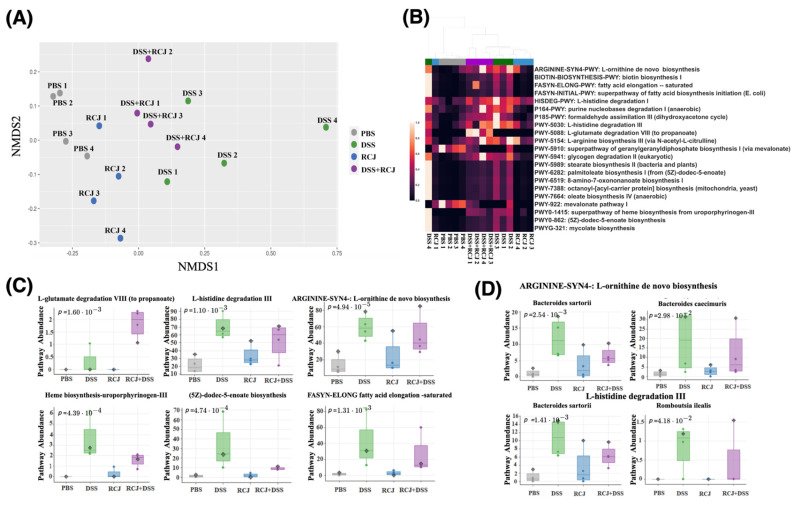
Effect of RCJ on biochemical pathways. (**A**) NMDS plots for the biochemical pathways in the microbiota. (**B**) Differentially abundant biochemical pathways across treatment groups. (**C**) Top significantly regulated pathways that are beneficial for the colon epithelium health. (**D**) Selected organism-specific pathways with significantly differential abundance. *Bacteroides sartorii* and *Bacteroides caecimuris* are the two main species associated with arginine synthesis and L-histidine degradation.

NMDS plots for the CPM-normalized abundance of the biochemical pathways detected from the metagenomic data showed a similar trend to that observed in the taxonomy analysis. RCJ restored the functional profiles of the microbiome closer to those of the control condition (Figure 6A). Functional analysis of the microbiome revealed several pathways significantly associated with the DSS group. A comprehensive list of associated pathways is provided (Supplementary File S2 and Supplementary File S1 Figure S5). Here, several significant pathways were highlighted that were found to be relevant to colitis: arginine synthesis, biotin biosynthesis, long-chain fatty acid biosynthesis (oleate biosynthesis and 5Z-dodec-5-enoate biosynthesis), heme biosynthesis, and L-histidine degradation. With RCJ treatment, these pathways were brought back closer to control (Figure 6B). On the other hand, arginine synthesis and L-histidine degradation, which were upregulated in the DSS group compared with the control, remained upregulated in the RCJ + DSS group compared with the control group (Figure 6C). The pathway for L-glutamate degradation to propionate was one of the very few pathways that showed an enrichment trend only in the RCJ + DSS group (Figure 6C). *Bacteroides sartorii*, and *Bacteroides caecimuris* are the two species associated with arginine synthesis and L-histidine degradation (Figure 6D).

### 2.7. RCJ Is Reversing the Dysregulation of Immunological Responses in DSS-Induced Colitis Mice

**Figure 7:**
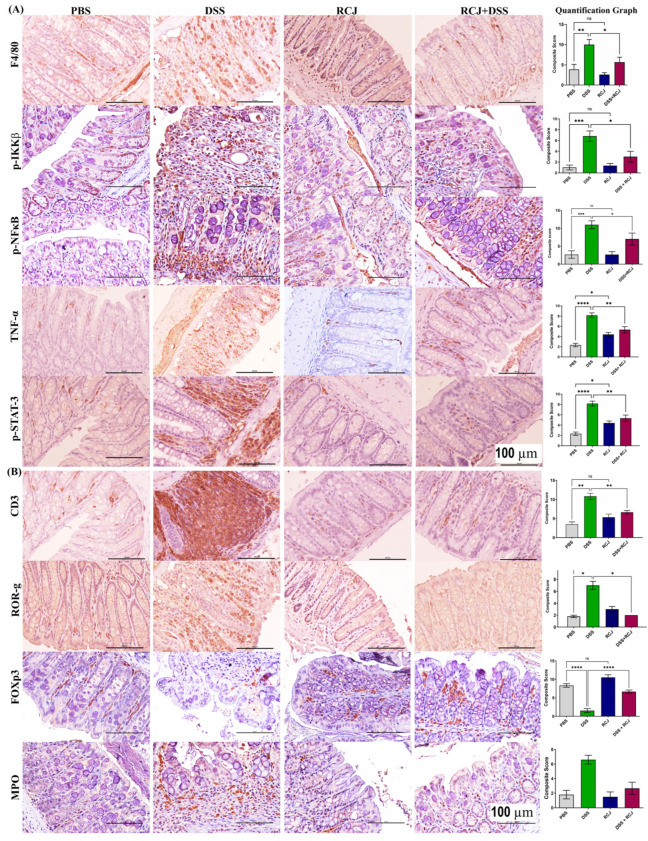
RCJ reversing the dysregulation of immunological responses in DSS-induced colitis mice. (**A**) IHC staining for F4/80 (total macrophages), and to check inflammation stained for p-IKKβ, p-NF-κB, and p-STAT (**B**) IHC staining for T cell marker panel with CD3, RORγ, FOXp3, and MPO for the neutrophils. All values represent the means ± SD; error bars in the histograms are the standard error of the mean. Scale bars represent 100 µm for the IHC. Statistical significance was determined using one-way ANOVA, followed by the Tukey test. ns: non-significant, * *p* ≤ 0.05, ** *p* ≤ 0.01, *** *p* ≤ 0.001, **** *p* ≤ 0.0001.

The release of pro-inflammatory cytokines was reduced following treatment with RCJ. We then aimed to elucidate the role of immune cells in the regulation of these events. First, we examined two central immune cell populations, macrophages and T cells, stained for F4/80 and CD3 markers. Both populations were increased in the DSS group. Interestingly, the DSS + RCJ both had significantly (*p* < 0.05; *p* < 0.01) fewer macrophages and T cells (Figure 7A,B). 

Due to the loss of intestinal barrier function in DSS-treated animals, coupled with the loss of tight junction proteins and epithelial cell damage, leakiness resulted. This allowed microbial translocation (lipopolysaccharides (LPS) from gram-negative bacteria) with the release of other bacterial endotoxins from the colonic lumen to the lamina propria. These events triggered the maturation of T helper 17 (Th17) and recruitment of neutrophils in the lamina propria, which aggravated oxidative stress and secretion of G-CSF, as confirmed by our cytokine array analysis. Additionally, G-CSF stimulates the bone marrow to produce more neutrophils. Thus, ROR-γ, a specific marker for the Th17 T cell subtype, and an MPO-specific neutrophils marker, were stained. The DSS group had a higher ROR-g and MPO-expressing cell population than the DSS + RCJ group (Figure 7B), which signified (*p* < 0.05; *p* < 0.01) that RCJ treatment reduced Th17 T cell maturation and neutrophil recruitment. 

Previous studies have shown that colonic Foxp3^+^ regulatory T cells (Tregs), an anti-inflammatory subset of CD4^+^ T cells, maintain immune homeostasis [37]. Thus, we checked for the Treg population in the colonic region and found that the DSS group had a significantly lower Foxp3^+^ Treg cell population (Tregs (*p* < 0.001) than the DSS + RCJ group, suggesting that RCJ could have an immunoregulatory effect (Figure 7B) that secretes IL10, an anti-inflammatory cytokine.

Furthermore, lipopolysaccharide (LPS) can cause inflammation by activating TLRs on macrophages present in the lamina propria. This, in turn, activates the NF-κB pathway, which is a ubiquitous transcription factor that is well characterized and is a primary mediator of the inflammatory response and increases pro-inflammatory cytokine levels TNF-α, IL6, IL-1β, and COX-2 levels in these macrophages. Increases in TNF-α act as autocrine signaling for the activation of NF-κB, and an increase in IL6 causes the activation of the STAT3 pathway. p-IKKβ, pNF-κB, TNF-α, and pSTAT3 levels were increased in the DSS group, whereas they were significantly reduced in the DSS + RCJ group (*p* < 0.05, *p* < 0.05, *p* < 0.01; *p* <0.01) (Figure 7A). Overall, RCJ treatment inhibited the recruitment of immune cells and lowered the degree of inflammation in DSS-induced colitis.

**Figure 8:**
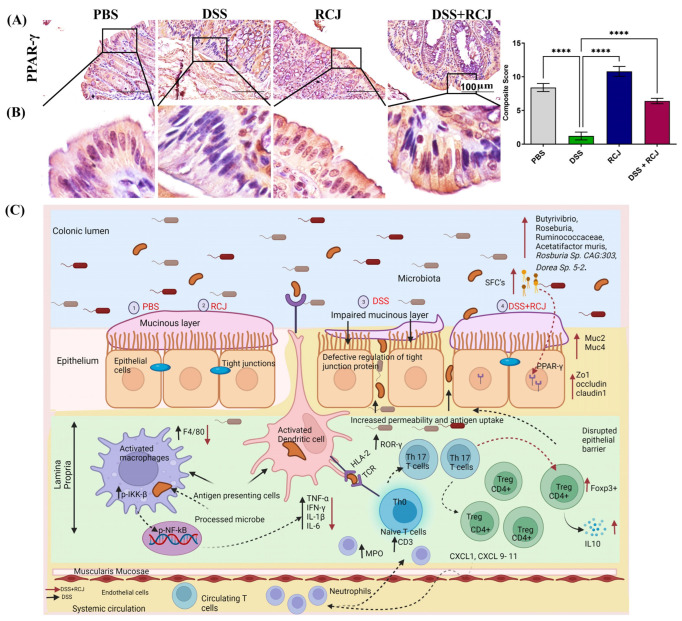
(**A**) IHC staining for anti-inflammatory mediators PPAR-γ indicative of butyrate presence. Scale bars represent 100 µm for the IHC. Statistical significance was determined using one-way ANOVA, followed by the Tukey test. **** *p* ≤ 0.0001. (**B**) Zoom image of PPAR-γ expression. (**C**) Schematic model showing the mechanism by which RCJ alleviated DSS-induced colitis. Intestinal microbiota, oxidative stress, inflammation, and barrier integrity are all affected. RCJ treatment changed the gut microbiota by enriching bacteria such as *Butyrivibrio, Roseburia, Ruminococcaceae*, *Acetatifactor muris*, *Rosburia* Sp. *CAG:303*, *Dorea* Sp. *5-2,* which subsequently led to increased production of SCFAs such as butyrate, which was evidenced by increased expression of PPAR-γ leading to a cascade of events, including anti-oxidative, anti-inflammatory, and barrier-protective responses. Ultimately, intestinal epithelial homeostasis is attenuated, and colitis is attenuated.

Our data analysis also showed that an increased *Firmicutes* and *Bacteroidetes* (F/B) ratio indicates high SCFA production in the RCJ + DSS group compared to that in the DSS group [38,39]. This was also evidenced by increased PPAR-γ expression. Microbiota-derived SCFAs in the gut help regulate the host immune response, maintain the intestinal mucosal barrier, and balance the intestinal microbiota [40,41]. PPAR-γ is a crucial anti-inflammatory mediator that can sense butyrate and is expressed at high levels in the colon. Butyrate is an important SCFA known to modulate immune responses [42]. To further determine whether RCJ ameliorates colitis via microbiota-derived SCFAs/PPAR-γ, PPAR-γ expression was investigated in the colon. PPAR-γ levels were dramatically reduced in the DSS group compared to those in the DSS + RCJ group (Figure 8A,B).

## 3. Discussion

Recently, several dietary supplements have emerged as promising therapeutic interventions for IBS and its associated diseases. In the current study, we investigated the effect of RCJ in a DSS-induced colitis mouse model. This model is currently widely used to study IBD because of its resemblance to human ulcerative colitis. In humans, IBD is linked with gut dysbiosis [43]. Consumption of RCJ is inversely correlated with inflammation and oxidative stress owing to a group of bioactive compounds present in RCJ [26,27,31]. However, the active RCJ components that regulate gut microbiota to confer anti-inflammatory function and intestinal homeostasis are unclear.

In this study, we focused on ameliorating DSS-induced colitis by RCJ treatment while understanding the effects of the gut microbiome. Oral administration of RCJ markedly ameliorated DSS colitis, as demonstrated by reduced body weight loss and higher survival rates. In addition, mice recovered when receiving RCJ during the resting period after the first cycle of DSS. The significant features of the DSS-induced colitis model are short colon length, high blood levels in feces, increased diarrhea, and determination of DAI. Considering that the reduction in colon length is a classic indicator of experimental colitis, the treatment group that received RCJ followed by DSS treatment did not show a colon length-shortening effect [44]. Our results showed that supplementation with RCJ significantly reversed these critical features of colitis. This is consistent with previous reports where Rhein, *Ilex kudingcha*, and Pu-erh Tea extract alleviated DSS-induced colitis [43,45,46]. DAI is an indicator of disease severity and is comparable to the human clinical representation of IBD. Treatment with RCJ reduced disease severity in the DSS group, indicating that RCJ might function as a prebiotic. This might provide a novel and over-the-counter effective colitis prevention and therapeutic strategy.

To maintain intestinal homeostasis, intestinal barrier function and permeability are vital, as shown by experimental and clinical data [47]. Intestinal epithelial cells, the mucus layer, and tight junction proteins mainly contribute to intestinal permeability. H&E staining revealed inflammatory cell infiltration, intestinal architectural changes, loss of villi and necrosis of the intestinal surface, reduced cell proliferation, and increased apoptosis in the DSS-treated mice. 

Moreover, RCJ significantly attenuated DSS-induced oxidative stress by enhancing the expression of antioxidant enzymes, such as SOD and GPX4 and reducing 4 hydroxynonenal expression, which is a crucial mediator of oxidative stress-induced cell death in the colon, which is corroborated by previous studies [48].

To determine the effects on the protective mucin layer, PAS staining for acid and neutral mucin showed significant loss of mucins in the DSS-treated group. H&E staining also showed that the PBS control group displayed intact colonic mucosa, crypts, stroma, and submucosa. There was no inflammatory cell infiltration in the submucosa or ulceration. In contrast, the DSS group showed severe epithelial cell damage, shortening and hyperplasia of crypts, submucosal edema, inflammatory lesions, severe inflammatory cellular infiltration in the submucosa, and macroscopic spaces between the crypts. However, RCJ treatment ameliorated the colon inflammatory symptoms and intact surface epithelium, leading to less inflammatory cell infiltration in crypts and only mild submucosal edema. This condition of the colon resembled closely that of PBS mice. PAS and Alcian blue staining showed that the DSS-treated group had a significant reduction in the thickness of colonic epithelial mucosa, which was healed to near-normal thickness.

Recent studies have suggested that mucins initiate inflammatory bowel disease, leading to cancer progression [49]. In contrast, other groups retained mucin with increased expression of MUC2 and MUC4, suggesting that RCJ plays a protective role against inflammatory colitis, as determined in a mouse model [50]. Mucins are important in protecting the gastrointestinal tract and eliminating bacterial toxins. 

From our initial H&E data, we found disruption of the crypt structure in the DSS-treated group, which was improved in the DSS + RCJ group. Thus, to understand how RCJ enhances intestinal barrier function, the tight junction proteins Claudin, Occludin, and ZO-1 were assessed, as they play a vital role in maintaining intestinal barrier function and regulating cellular permeability. Decreased expression of tight junction proteins is observed in most IBD cases. Several factors, including dietary components, gut microbiota, and cytokines, regulate intestinal tight junction proteins [51]. Previous reports have shown that ZO-1 downregulation may be one of the causes of ineffective mucosal healing in IBD patients [52]. 

The gut microbiota modulates the immune system by releasing several mediators such as short-chain fatty acids (SCFAs). These mediators, released by immune cells, can induce tight junction dysfunction. Our results also showed that supplementation with RCJ enhanced the expression of tight junction proteins. This finding aligns with a previous report where ZO-1 expression was improved in mouse intestinal epithelial cells when treated with hyaluronan [53]. 

Immune cells secrete cytokines, which are small peptides. Cytokines act as vital pathophysiological regulators that govern the occurrence and development of inflammation leading to IBD [54]. The DSS-mediated stimulation of pro-inflammatory cytokines highlights the role of the innate immune system in the development of IBD [55]. Our study demonstrated a DSS-induced increase in pro-inflammatory cytokines, while RCJ administration attenuated this inflammatory response. RCJ alleviated colonic inflammation by reducing inflammatory cytokines, such as TNF-α, IL-6, IL-1β, and IFN-γ, and increasing IL-10 in the plasma of DSS-treated mice. Proinflammatory cytokine genes have a binding site for NF-κB and regulate transcription [56]. In addition, DSS treatment causes damage to the colon epithelial cells and leads to tissue inflammation due to the accumulation of nitric oxide (NO) generated by iNOS. RCJ treatment reduced iNOS expression and alleviated inflammatory effects.

Furthermore, the elevated COX-2 in DSS was due to loss of gut permeability, as the cell wall component LPS, of gram-negative bacteria, can stimulate epithelial cells to promote the production of COX-2 and cause inflammation [57]. However, this was reversed by RCJ supplementation. The RCJ-treated group showed reduced macrophage (F4/80) and increased T-reg (Foxp3) cell infiltration, crypt destruction, and ulcer formation due to reduced pro-inflammatory cytokines (such as TNF-α, IL-6, IL-1β, and IFN-γ) and increased anti-inflammatory IL-10 in serum. These results are highly consistent with previous reports [58]. 

Gut microbiota contributes to immune responses and intestinal permeability through a variety of enzymes and metabolites. This study found that DSS treatment results in microbiome shifts from both taxonomic and functional perspectives and that RCJ can be used to mitigate this change. The low abundance of *Firmicutes* and high abundance of *Bacteroidetes* is a classic signature of microbiota dysbiosis in DSS. RCJ supplementation reversed these effects. 

Nutraceuticals are attracting increasing attention worldwide as potent therapeutic agents for IBD, owing to their fewer side effects. These active compounds and gut bacteria have a commensal relationship. The active compounds module the gut microbiome, and the microbiome can also alter the bioactive compounds’ signature [59].

SCFAs (acetate, butyrate, and propionate) are produced in the colonic lumen, mainly by the fermentation of dietary fiber by the gut microbiota. These SCFAs are vital for pathophysiological colonic events [60]. For example, SCFAs are known to alleviate colitis by reducing the production of pro-inflammatory cytokines, thus blocking the NF-κB and STAT 3 signaling pathways. 

Taxonomic analysis of our metagenomic data suggests that several taxa known for SCFA production activity were enhanced in the DSS + RCJ treatment groups. These SCFAs act as a significant energy source for colonic mucosal cells and are essential regulators of gene expression during crucial events such as apoptosis, differentiation, and inflammation. These microbiota-derived SCFAs prevent infections by modulating the systemic immune response and enhancing the intestinal mucosal immune barrier [61]. Thus, SCFA-producing bacteria act as probiotics and play vital roles in various biological functions.

Deficiency in the butyric acid-producing genera that reside in the human intestinal tract has been linked to disease states [34,62,63,64]. Our data showed that butyric acid-producing gut bacterial populations were destroyed by DSS treatment, while RCJ effectively recovered them in the RCJ and RCJ + DSS groups. More specifically, *Dorea* sp. 5-2 species stands out, as it has an undetectable population in the DSS group, while its abundance in RCJ and RCJ + DSS was closer to that in the control group. This suggests an interesting hypothesis about the mechanism by which RCJ improves the microbiome and inflammation status by enriching this specific microbial pathway related to SCFA production in an unhealthy microbiome. In contrast, DSS treatment promoted the population of *Bacteroides sartorii*. Our pathway analysis data indicated *Bacteroides sartoriis*’ role in increasing the abundance of the arginine biosynthesis pathway. This suggests that *Bacteroides sartorii* caused an imbalance in arginine levels in the DSS group. 

The high levels of butyrate-producing bacteria found in the RCJ + DSS group controlled PPAR-γ expression (an anti-inflammatory mediator). Under DSS conditions, PPAR-γ levels were significantly low. To further validate the effect of RCJ on the colitis model via microbiota-derived SCFAs/PPAR-γ, the RCJ + DSS treatment group showed increased expression, suggesting IBD might be correlated with PPAR-γ deficiency. This trend was described in previous reports where phloretin enhanced PPAR-γ expression in DSS-induced mice [65]. PPAR-γ is also known to inhibit the nuclear factor-κB (NF-κB) and MAPK pathways. Regulation of these key signaling networks inhibits the production of cytokines and chemokines, which reduces the buildup of inflammatory cells [66]. Based on our current findings, SCFAs derived from microbiota ameliorated colitis in mice by increasing PPAR-γ expression. Butyrate may also affect the histone acetylation of gut CD4+ T cells to epigenetically control the production of genes necessary for Treg cell induction. Previous studies have shown that colonic Foxp3^+^ regulatory T cells (Tregs), which are an anti-inflammatory subset of CD4^+^ T cells, maintain immune homeostasis [37]. Our results showed that the Foxp3^+^ Treg cell population was increased in the DSS + RCJ group, which might be crucial for immune cells that secrete IL10, a vital anti-inflammatory cytokine [67].

A mechanistic understanding of dietary supplementation and gut microbial metabolism is important, as microbiome-derived metabolites can lead to divergent physiology and aid in gut homeostasis [68]. Overall, RCJ significantly restored the imbalance introduced by DSS. However, it is not possible to directly infer whether the microbiome is only affected by DSS treatment or whether DSS plays a role in initiating or influencing pathology. An increasing number of studies indicate the importance of fatty acids as pro- or anti-inflammatory agents [69,70]. Early metabolomics studies on IBD have determined increased levels of long-chain fatty acids in patients with IBD [71]. Specifically, octanoate and decanoate have been shown to accumulate in considerable amounts in pathological tissues, leading to impairment of the functioning of mitochondrial respiratory complexes [72]. This directly contradicts the supposed role of short-chain fatty acids as a readily available energy source for epithelial cells [73]. Interestingly, glutamate degradation to propionate was elevated in the DSS + RCJ group. This indicates an increase in the SCFA producer population. This suggests a link between colitis and microbiome metabolism. During colitis, the production of LCFAs is favored, while treatment with RCJ favors SCFA over LCFAs. The reason for this selective behavior is unclear. Oxidative stress induced by DSS treatment could be an initiator that shifts microbiome metabolism. This is intensified when DSS treatment and induced colitis mucosal permeability are compromised, providing a higher concentration of available oxygen [74]. The higher abundance of heme biosynthesis pathways in the DSS group also supports this hypothesis. This suggests that reducing oxidative stress by RCJ treatment might be an important mechanism for the regulation of the gut microbiome metabolism. The observed increase in glutamate degradation for propionate production in RCJ-supplemented mice also suggests a metabolic change with RCJ treatment after DSS treatment. DSS treatment also caused a change in microbial metabolism of several amino acids compared with that in the control group. Arginine biosynthesis genes were present in higher abundance in the DSS group. Higher concentrations of arginine can produce nitrite oxide, which is increased in patients with UC [75]. The same trend was observed for L-histidine degradation pathways in the DSS treatment groups. A lower available histidine concentration is associated with a higher risk of relapse in UC patients [76]. 

In conclusion, DSS treatment resulted in decreased abundance of the SCFA-producing bacteria population and promoted abundance in the pathogenic bacteria population. Additionally, this population shift is accompanied by dysregulation of the gut microbiome metabolism, especially fatty acid and amino acid metabolism.

## 4. Methods

### 4.1. Pulsed Electric Field-Assisted Extraction (PEF) of Red Cabbage Juice (RCJ)

Red cabbages were obtained from the local market and processed by using PEF with the processing parameters of 1.2 kV/cm, 2 μF, 25 pulses, 3.43 kJ/kg. The samples of mash derived from PEF-treated red cabbages were mechanically pressed (450 N, 9 min). The extract of red cabbage juice (RCJ) was stored at −80 °C and used for further biochemical assays. The pH was measured using a pH meter.

### 4.2. Phenolic Compounds Quantification of RCJ

The initial bioactivity of PEF-treated RCJ was analyzed for total phenolic compounds and total anthocyanin concentrations. An aliquot of each of the samples frozen at −80 °C was used for determination of the same bioactivity components to test the effects of freezing. The total iron-reactive phenolic compound concentration in RCJ was quantified by UV-Vis spectrophotometry using a previously published method [77]. RCJ anthocyanin concentration was analyzed using a 375 1260 Infinity II HPLC (Agilent Technologies, Santa Clara, CA, USA) with a reserved-phase column (Li Chrospher 100-5 RP18 250 × 4.0 mm, Agilent Technologies), DAD (Agilent 1260 377 Infinity II DAD WR) and fluorescence detector (FLD) (Agilent 1260 Infinity II FLD Spectra) as in previous reports [77]. Ammonium dihydrogen phosphate (50 mM) was used as the mobile phase at pH 2.6 (mobile phase A), 20% (*v*/*v*) mobile phase A in acetonitrile (mobile phase B), 0.2 M ortho-phosphoric acid in water, pH 1.5 (mobile phase C). The temperature of the column was maintained at 40 °C at a flow rate of 0.5 mL/min. The sample supernatant (20 μL) was injected. Monomeric phenolics were identified and quantified at different wavelengths: 280 nm for gallic acid, 360 nm for flavonols, 316 nm for hydroxycinnamic acids, and 520 nm 385 for anthocyanins. FLD was used to detect and quantify (+)-catechin and (−)-epicatechin at an excitation wavelength of 276 nm and an emission wavelength of 316 nm. Cyanidin-3-O-glucoside was used as a standard to quantify anthocyanins present in RCJ. 

### 4.3. Sugars and Organic Acids Were Quantified in RCJ

Sugars and organic acids were quantified in the RCJ using high-performance liquid chromatography (HPLC) (1200 series, Agilent Technologies) with a diode-array detector (DAD) and refractive index detector (RID) (Agilent 1200 series). Bio-Rad Aminex HPX-87H and Bio-Rad fermentation monitoring columns with H+ guard cartridges were used, and a sample of 10 μL was injected. Sulfuric acid (5 mM) was used as the mobile phase with a flow rate of 0.65 mL/min for 35 min. Malic acid detection was performed at 210 nm using DAD. Other residual sugars were detected by using RID (cell temperature of 55 °C). Commercial standards from Bio-Rad were used to obtain calibration curves for each compound.

### 4.4. Animal, Induction of Colitis, Treatment and Sample Collection

To evaluate the protective role of RCJ in DSS-induced colitis, we employed 60 C57Bl6 mice in this study. Seven- to eight-week-old specific pathogen-free (SPF) male and female C57BL/6 J mice at an equal ratio, housed in groups of five mice in one standard cage in an animal facility at the University of Nebraska Medical Centre, followed a 12 h light and 12 h dark cycle at 22 °C. Colitis was induced by administering 3% DSS (36–40 kDa) through drinking water. 

One week after acclimatization of the mice in the animal facility, the mice were randomly divided into 2 treatments, 30 mice in each treatment group. Group 1 was given PBS (200 µL) (n = 30), whereas group 2 was given RCJ (200 µL) (n = 30) by oral gavage every day for eight weeks. After eight weeks of treatment, the above groups were further divided into two groups. The PBS group was further divided into two sub-groups. The first subgroup was given PBS alone (n = 15) and the other subgroup was given PBS + 3% DSS (n = 15) treatment. Similarly, the RCJ group was divided into two subgroups, the first subgroup given RCJ alone (n = 15) and the other subgroup given 3%DSS + RCJ (n = 15). As described previously, the animals were provided with free access to water supplemented with or without 3% DSS after the 8th week for two cycles of 3% DSS treatment [the DSS treatment was given for 1 week (9th week), then followed by one week of recovery (10th week) and again followed by 3% DSS for 1 week (11 week)] [78]. During DSS treatment, RCJ and PBS was given as an oral gavage for these three weeks. Mice were euthanized by CO_2_ asphyxiation, followed by cervical dislocation at the end of the experiment.

Body weight was measured daily for the entire duration of the study. The disease activity index (DAI) was calculated to assess the severity of the colitis by combining scores of body weight loss, diarrhea of the stool, and the extent of blood in the feces. The mice were sacrificed with CO_2_ at the end of the 11th week, the length of the colon was measured, and cecal and colon contents (fecal samples) from all mice were collected aseptically and flash-frozen in liquid nitrogen and stored at −80 °C for future analysis. Blood samples were collected from each mouse and plasma was obtained by centrifugation (3000× rpm for 15 min at 4 °C) and then stored at −80 °C for future measurement of the cytokine. The colon and cecum were flushed with PBS and then fixed in 10% formalin for subsequent histology analysis. At the same time, part of the colon and cecal tissue was washed with PBS and snap-frozen in liquid nitrogen for future analysis.

The blood and other organs were collected and stored. Our full animal protocol can be found at PCT ID:PCTE0000439. (https://preclinicaltrials.eu/database/view-protocol/439 accessed on 1 December 2023) 

### 4.5. Disease Severity

Diarrhea, blood in feces scores, and mouse body weight were monitored throughout the experiment. Diarrhea scores and blood in feces scores were recorded daily and scored from 0 to 3 (absent, mild, moderate, and severe). To assess disease severity, the disease activity index (DAI) was calculated, as previously described [54]. Briefly, the DAI was calculated as a combination of weight loss, diarrhea, and bleeding scores, resulting in a colitis score of 0–10 (unaffected by severe colitis). Body weight scores were calculated based on the change in body weight from the original weight (≥ 0% = 0; −5% to 0% = 1; −10% to −6% = 2; −15% to −11% = 3; and < −15% = 4). This bodyweight score was combined with diarrhea and blood in feces scores to calculate DAI. 

### 4.6. Cytokine Analysis

Pro-inflammatory cytokines, such as IFN-γ, TNF-α, IL-1α, IL-1β, IL-1ra, IL-5, IL-6, IL-10, IL-12, IL-13, IL-17, and IL-23, are associated with IBD [79,80]. These cytokines were analyzed using a cytokine array kit according to the manufacturer’s protocol (Proteome Profiler Mouse Cytokine Array Kit, Panel A; R&D Systems, Minneapolis, MN, USA). Plasma samples (100 μL/group) were pooled within each group, with five samples in the PBS group and six in the other groups. The results were quantified by analyzing the pixel density of each spot on the developed film using the Image J software (http://rsb.info.nih.gov/ij accessed on 1 December 2023).

### 4.7. Histology

Colon tissue samples harvested from the mice were fixed in 10% buffered formalin. The samples were then processed and embedded in paraffin blocks. From each block, 4 μm sections were prepared. The prepared colon tissue sections were stained with hematoxylin and eosin (H&E) and periodic acid-Schiff (PAS) for all treatment groups (*n*= 6/group). The H&E-stained tissues were scored based on inflammatory cell infiltration (0–3) and changes in intestinal architecture (0–3). These two scores were summed to obtain a final score of 6. The PAS-stained tissues were analyzed by counting the number of PAS-positive cells. The number of cells per crypt is reported, with a minimum of 20 crypts in each section. 

### 4.8. Terminal Deoxynucleotidyl Transferase dUTP Nick End Labeling (TUNEL) Assay

A commercially available kit (ab206386 Abcam, Boston, USA) was used to detect apoptotic cells in mouse colon tissues. Briefly, 5 mm-thick tissue sections were deparaffinized in xylene and further dehydrated with a series of graded alcohol, followed by proteinase K treatment and 3% H_2_O_2_ treatment to inactivate endogenous peroxidases in the cells. Biotin-labeled deoxynucleotide incorporation in apoptotic cells, catalyzed by terminal deoxynucleotidyl transferase (TdT), was detected by incubation with streptavidin-horseradish peroxidase (HRP) conjugate. Signals were detected using 3,3′-diaminobenzidine (DAB) substrate, and sections were counterstained with methyl green. Positive and negative control tissues, treated with DNase I and water instead of TdT, were used for comparison.

### 4.9. Immunohistochemistry/Immunofluorescence

For immunohistological analysis, the prepared tissue sections were heated overnight and then deparaffinized in xylene (2X 5 min). The sections were then hydrated using graded alcohol. Then, citrate buffer (0.01 M, 95 °C, pH 6.0) was used for antigen retrieval. 0.3% H_2_O_2_ in methanol for 30 min was used to quench the endogenous peroxidase activity. After PBS washing, sections were blocked with 2.5% horse serum (ImmPRESS kit; Vector Labs, Burlingame, CA, USA) for 2 h. Next, sections were incubated with primary antibodies and kept at 4 °C overnight. After washing with PBS containing 0.01% Tween 20 (PBST), the sections were incubated for 30 min with a secondary antibody (peroxidase-labeled anti-mouse/anti-rabbit IgG (ImmPRESS kit, Vector Labs, Burlingame, CA, USA)). The sections were washed with PBST (3X, 5 min) and developed using a DAB substrate kit (Vector Laboratories, Burlingame, CA, USA). Counterstaining was performed with hematoxylin. Graded alcohol was used to dehydrate the slides, followed by xylene (2X, 5 min). After drying, the slides were mounted in toluene and imaged [81,82]. The following antibodies from various vendors were used for the immunohistochemistry study: anti-Ki 67 (Abcam, ab15580); Anti-SOD1 (CST 65778, Boston, MA, USA); Anti-GPX4 (ab125066, Abcam, Boston, MA, USA); Anti-4-Hydroxynonenal (R&D Systems; Biotechne, MAB3249, Minneapolis, MN, USA); Anti-MUC2 (Abcam, ab272692, Boston, MA, USA); Anti-MUC4 (Santa Cruz, SC-33654, Dallas, TX, USA); anti-claudin 1 (Proteintech, 13050-1-AP, Rosemont, IL, USA); anti-occludin (Proteintech, 66378-1-Ig); Anti-F4/80 (Abcam, ab300421, Boston, MA, USA); anti-pIKKβ (CST, 36214SF, Boston, MA, USA); anti-pNF-kB (Abcam, ab131100, Boston, MA, USA); anti-TNF-α (Abcam, ab1793, Boston, MA, USA); Anti-pSTAT3 (CST, 9145, Boston, MA, USA); Anti-CD3 (CST, 78588, Boston, MA, USA); Anti-RORγ (Abcam, ab207082, Boston, MA, USA); Anti-FOXp3 (CST, 12653, Boston, MA, USA); Anti-MPO (Proteintech, 22225-1-AP); Anti-Ppar g (Proteintech, 16643-1-AP, Rosemont, IL, USA). All immunostained slides were analyzed by a pathologist. Slides were assessed based on a previously reported method [83]. For immunofluorescence staining, antibodies against ZO-1 (Proteintech, 21773-1-AP, Rosemont, IL, USA), iNOS (Proteintech, 22226-1-AP, Rosemont, IL, USA), and COX-2 (Proteintech, 66351-1-Ig, Rosemont, IL, USA) were used. Appropriate secondary antibodies conjugated to Alexa Fluor 488 or 594 (Vector Laboratories, Newark, CA, USA) were added at 1:200 dilution, and nuclei were counterstained with 4′,6-diamidino-2-phenylindole (DAPI) (Vector Laboratories H-1800, Burlingame, CA, USA).

### 4.10. Metagenomic Sequencing

Mouse fecal samples were collected before and at the end of the study from all groups and stored at −80 °C until fecal microbial DNA isolation. Microbial DNA was isolated from mouse fecal samples (QIAamp Power Fecal Pro DNA Kit; Qiagen, Hilden, Germany). At the end of the experiment, samples were analyzed from three animals in each group. The DNA samples were purified using Zymo Spin columns (Zymo Research Irvine, CA, USA). Libraries were prepared using a Nextera Flex DNA Kit (Illumina, San Diego, CA, USA). The concentrations of the libraries were measured in a Qubit30 using a high-sensitivity kit. The quality (size distribution) of the libraries was assessed using an Agilent 2100 Bioanalyzer. The libraries were pooled at an equimolar ratio and denatured in the presence of NaOH. The loading concentration was 1.5 pm, and sequencing was performed in Nextseq NS500 (Illumina, San Diego, CA, USA). A 150-base paired-end run and high-output flow cell were used. Sequencing was performed using Base space (Illumina).

### 4.11. Taxonomic and Functional Analysis

FASTQ files for each sample were aligned against the full NCBI NR database reference genome using DIAMOND [84]. The alignment files were processed using MEGAN 6 [85]. Taxonomic abundances, which were generated based on the output from the MEGAN analysis, are represented in bar graphs. The groups were compared using the linear discriminant analysis (LDA) effect size (LEfSe) method [86]. An LDA score of > 2 was used to identify features that significantly discriminated among groups. A cladogram was generated based on differential abundance values using LEfSe. Box plots were generated using Statistical Analysis of Metagenomic Profiles (STAMP) analysis [87]. The YAMP pipeline V 0.9.5.3 [88] was used to profile the metabolic functions of the samples, directly mapping the short reads to the reference databases of HUMAnN 3 and MetaPhlAn 3. YAMP leverages FastQC [89] and MultiQC [90] for quality check, BBDuk [91] for trimming, BBWrap for decontamination, MetaPhlAn [92] for taxonomic profiling, and HUMAnN [92] for functional profiling of metagenomic raw reads. Raw reads were decontaminated by removing reads mapped to the mouse genome with more than 97% similarity. Reads shorter than 70 bp were excluded. Additionally, the YAMP database of sequencing artifacts and adapters was used to trim the short reads using BBwrap. Trimmed reads were quality-checked, and all samples showed satisfactory quality and number of reads in each sample. HUMAnN 3 in the YAMP pipeline was used for the functional profiling of quality-checked reads. The outputs of HUMAnN were normalized to count per million (CPM). Multivariable association discovery in population-scale meta-omics studies (MaAsLin2) [93] was used to extract the association between pathways and gene counts with different treatments (PBS, RCE, DSS, and RCE + DSS) using a linear mixed model. All statistics of pathway association with the treatment groups were extracted from the outputs of MaAsLin2. Any pathways or reactions associated with the sex of the mice were discarded from the final analysis. The significant results were filtered to exclude results with *q*-value > 0.05, or *p*-value > 0.05. Non-metric multidimensional scaling (NMDS) [94] in the Vegan R package v2.6-4 [95] was used to map pathway and taxonomy data to 2-dimensional space. Non-metric multidimensional scaling (NMDS) plots were generated using the ggplot2 R package [96]. The Vegan package in R was used to create alpha diversity measures for each sample. MultiQC output, the raw outputs and log files of YAMP, the YAMP configuration files that were used, and a Jupyter notebook providing the steps taken along with the parameters used in each step and the generated plots, are all available at (https://github.com/chan-csu/RCJ_Megtagenomics accessed on 1 December 2023).

### 4.12. Statistical Analysis

Statistically significant differences were analyzed using the Student’s *t*-test with a 0.05 significance level (*p* < 0.05). Linear mixed-effects models were used to analyze the changes in body weight and DAI scores over time. Treatment group, time, and treatment-by-time interactions were included in the model. The percent change in the body weight model was adjusted for the baseline weight. Pairwise comparisons were adjusted for multiple comparisons using Tukey’s method. The Kaplan–Meier method was used to estimate the survival curve from induced colitis, and survival times were calculated as the days from treatment initiation to death from colitis on the last study date. Animals that died from causes other than colitis were censored. Toxicity data were summarized descriptively over time using box plots. SAS software (version 9.4; SAS Institute Inc., Cary, NC, USA) was used for data analysis. 

## 5. Limitations

The current study provides valuable insights into the potential benefits of RCJ in mitigating colonic inflammation. It is also essential to acknowledge the limitations of the study. The study was of short-term treatment outcome, and long-term effects of RCJ were not thoroughly investigated. Diversity in experimental animals was not taken into the experimental plan. To explore further the observed taxonomic trend from a functional perspective in the future, we plan to conduct additional metagenomic studies alongside meta-transcriptomic datasets. These investigations are on the horizon. Additionally, we aim to quantify short-chain fatty acids (SCFAs) using mass spectrometry and elucidate the precise molecular mechanisms governing SCFAs. Furthermore, we also are currently identifying the key active compounds in RCJ that foster the growth of these beneficial bacteria.

## 6. Conclusions

In summary, RCJ attenuated DSS-induced colitis in mice by altering the gut microbiota and enriching SCFA-producing bacteria, such as *Butyrivibrio*, *Ruminococcaceae*, *Acetatifactor muris*, *Rosburia* Sp. CAG:303, *Dorea* Sp. 5-2. The pathway abundance showed proof of SCFAs (L-glutamate degradation to propionates). Bacteria such as *Bacteroides sartorii* and *Bacteroides caecimuris*, responsible for histidine degradation, were significantly reduced in the RCJ + DSS-treated group. Increased expression of PPAR-γ indicated the presence of SCFAs (butyrate) in RCJ-treated groups. PPAR-γ inhibits the activation of the NF-κB signaling pathway and reduces the expression of IL6, TNF-α, and iNOS, which are crucial for inflammation. Further, RCJ treatment also increased the Treg cell (FOXP3^+^) population along with IL10 expression. Thus, these changes in the gut microbiota subsequently led to increased gut barrier function, colon repair, and anti-oxidative effects, resulting in the attenuation of intestinal damage and colonic inflammation (Figure 8C). These findings provide novel insights into how RCJ ameliorates DSS colitis by modulating the gut microbiota. Nutraceuticals hold promise in the development of preventive and therapeutic strategies for IBD patients.

## Data Availability

The data reported in this paper are accessible in the NCBI Short Read Archive (SRA) under accession ID PRJNA944265 (https://www.ncbi.nlm.nih.gov/sra/?term=PRJNA944265) accessed on 28 August 2023. The original R scripts and data used for statistical analysis are available at GitHub (https://github.com/chan-csu/RCJ_Megtagenomics accessed on 1 December 2023). The animal complete protocol is submitted to https://preclinicaltrials.eu/database/view-protocol/439 accessed on 1 December 2023 with PCT ID: PCTE0000439.

## Supplementary Materials

The following supporting information can be downloaded at: https://www.mdpi.com/article/10.3390/ijms25010539/s1.

## Abbreviations

RCJ: Red Cabbage juice; IBD: Inflammatory bowel disease; UC: Ulcerative colitis; DSS: Dextran sulfate sodium; CDC: Centers for Disease Control and Prevention; SCFAs: Short-chain fatty acids; LCFAs: Long-chain fatty acids; TNF-α: Tumor necrosis factor alpha; GSLs: Glucosinolates; PEF: Pulsed electric field; ITCs: Isothiocyanates; UV: Ultraviolet; HPLC: High-performance liquid chromatography; DAD: Diode-array detector; FLD: Fluorescence detector; RID: Refractive index detector; IL (1–17): Interleukin (1–17); IFN-γ: Interferon gamma; TUNEL: Terminal deoxynucleotidyl transferase dUTP nick end labeling; HRP: Streptavidin-horseradish peroxidase; Ppar g: Peroxisome proliferator-activated receptor gamma; iNOS: Inducible nitric oxide synthase; COX-2: Cyclooxygenase-2; DAPI: 4′,6-diamidino-2-phenylindole; MUC 2: Mucin 2; MUC 4: Mucin 4; pSTAT3: Phospho Signal transducers and activators of transcription 3; RORγ: Retineic-acid-receptor-related orphan nuclear receptor gamma; MPO: Myeloperoxidase; LDA: Linear discriminant analysis; LEfSe: Linear discriminant analysis effect size; STAMP: Statistical Analysis of Metagenomic Profiles; CPM: Count per million; MaAsLin: Multivariable association discovery in population-scale meta-omics studies; NMDS: Non-metric multidimensional scaling; GC-MS: Gas chromatography mass spectrometry; SOD: Superoxide dismutase; 4-OH-enol: Hydroxynonenal; GPX4: Glutathione peroxidase 4; CXCL: C-X-C motif chemokine; Th17: T helper 17; UC: Ulcerative colitis; NF-κB: Nuclear factor kappa-B; MAPK: Mitogen-activated protein kinase; IKKβ: Inhibitor of nuclear factor kappa-B kinase; G-CSF: Granulocyte colony-stimulating factor; GM-CSF: Granulocyte-macrophage colony-stimulating factor; CD3: Cluster of differentiation 3; LPS: Lipopolysaccharide; TLRs: Toll-Like Receptors; DAI: Disease activity index; Tregs: T regulatory cells; Th17: T helper 17; IKKβ: Inhibitor of nuclear factor kappa-B kinase; W/V: Weight/volume; DAI: Disease activity index; ZO1: Zonula occludens-1; GI: Gastrointestinal; TNF-α: Tumor necrosis factor-alpha; ITCs: Isothiocyanates; SRB: Sulfate-reducing bacteria; NO: Nitric oxide; PEF: Pulsed Electric Field-Assisted Extraction; H&E: Hematoxylin and eosin; PAS: Periodic acid-Schiff; PBS: Phosphate buffer saline; NMDS: Non-metric multidimensional scaling.
