# Supplementary material for: Red Cabbage Juice-Mediated Gut Microbiota Modulation Improves Intestinal Epithelial Homeostasis and Ameliorates Colitis
Source: Int J Mol Sci. 2023 Dec 30;25(1):539. doi: 10.3390/ijms25010539 (PMC10778654; doi:10.3390/ijms25010539)
Supplement: Supplementary file 1 [file ijms-25-00539-s001.zip › Supplementry File S1.pdf]

Sample ID	pH	Brix	Glucose (g/L)	Fructose (g/L)	Citric acid (g/L)	Malic acid (g/L)	Unknown acid (g/L as malic acid eq.)
RCJ	6.42 ± 00.5	6.4± 0.2	18.2± 0.2	15.1± 0.2	0.8± 0.1	3.0± 0.3	34.2± 2.1

Total phenolic compounds and free anthocyanins content by spectrophotometry

Sample ID	Total phenolic compounds content (mg/L catechin eq.)	Free anthocyanins content (mg/L malvidin-3-glucoside eq.)
RCJ	382.5 ± 93.5	257.9 ± 3.1

Table S1. Bioactive compounds' retention during freezing and PEF treatment (A) Total phenolic compounds and free anthocyanins content by spectrophotometry

Sample	Free anthocyanins content mg/L Cyanidin-3-glucoside eq.	Total hydroxycinnamic acids content (mg/L caffeic acid eq.)	
**Red cabbage juice**	254.3 ± 23.6	55.6 ± 2.3	
Hypothesized anthocyanins in juice (based on literature and UV/visible spectrum)		% of total anthocyanins	**mg/L**
Cyanidin-3-diglucoside-5-glucoside		22.7	57.61
Cyanidin-3-(sinapoyl)-diglucoside-5-glucoside		2.5	6.34
Cyanidin-3-glucoside-5-glucoside		5.5	13.97
Cyanidin-3-(feruloyl)(feruloyl)-triglucoside-5-glucoside		1.9	4.88
Cyanidin-3-(sinapoyl)-triglucoside-5-glucoside		0.8	2.12
Cyanidin-3-(feruloyl)-diglucoside-5-glucoside		43.6	110.78
Cyanidin-3-(feruloyl)(feruloyl)-triglucoside-5-glucoside		3.3	8.38
Cyanidin-3-(sinapoyl)(sinapoyl)-diglucoside-5-glucoside		19.7	50.23

Table S2. HPLC-DAD method to determine the composition of monomeric polyphenols present in the RCJ.

* The red cabbage juice was rich in anthocyanins and identifying all the anthocyanins was established only based on the literature review and UV/vis spectrum. Therefore, the name anthocyanins has to be taken with caution.

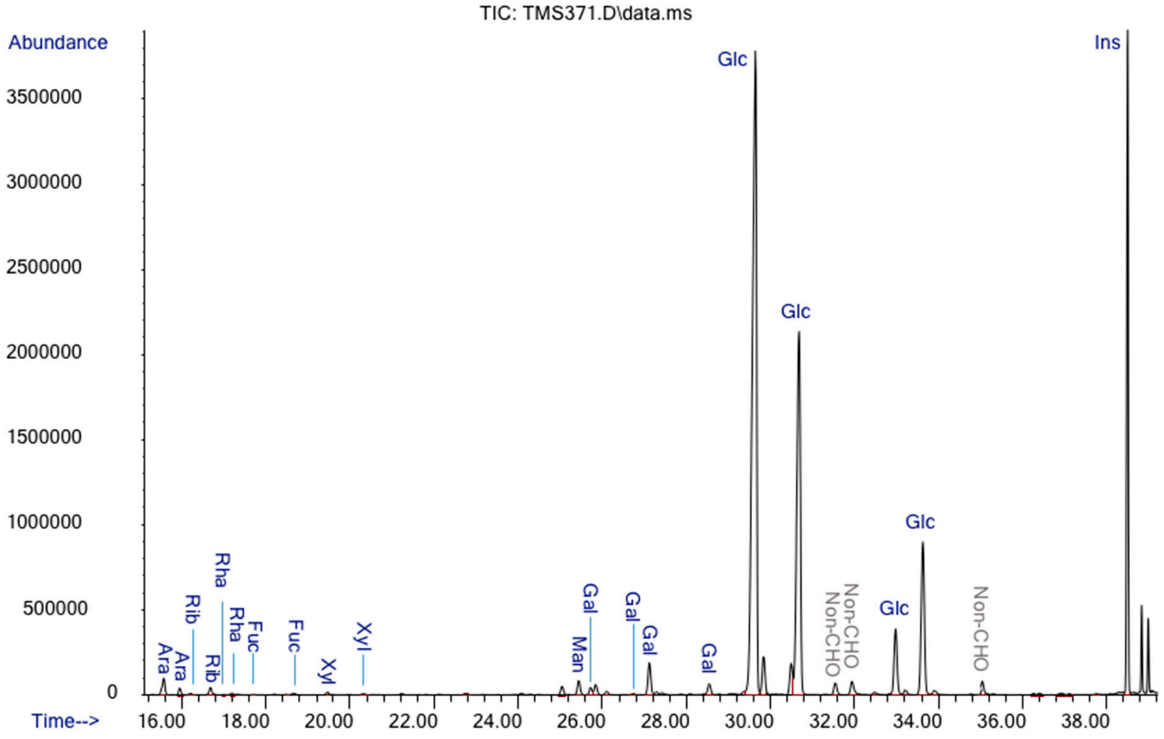

Figure S1. Glycosyl composition analysis by GC-MS of TMS derivatives of methyl glycosides. Shows Total ion chromatogram for the TMS residues from the cabbage juice. "Non-CHO" = non-carbohydrate.

Table S3. Calculated Mol % of monosaccharides and Total Carbohydrate percentage by weight for the sample.

Glycosyl residue	Cabbage Juice	
	Mass (µg)	Mol %[1]
Arabinose (Ara)	1.6	2.4
Ribose (Rib)	0.6	0.9
Rhamnose (Rha)	0.1	0.2
Fucose (Fuc)	0.08	0.1
Xylose (Xyl)	0.3	0.4
Mannose (Man)	1.0	1.3
Galactose (Gal)	3.5	4.5
Glucose (Glc)	70.4	90.1
SUM	77.6	99.9
Total Carbohydrate % by weight	19.4%	

[1]Values are expressed as mole percent of total carbohydrate. The total Mol % may not add to exactly 100% due to rounding.

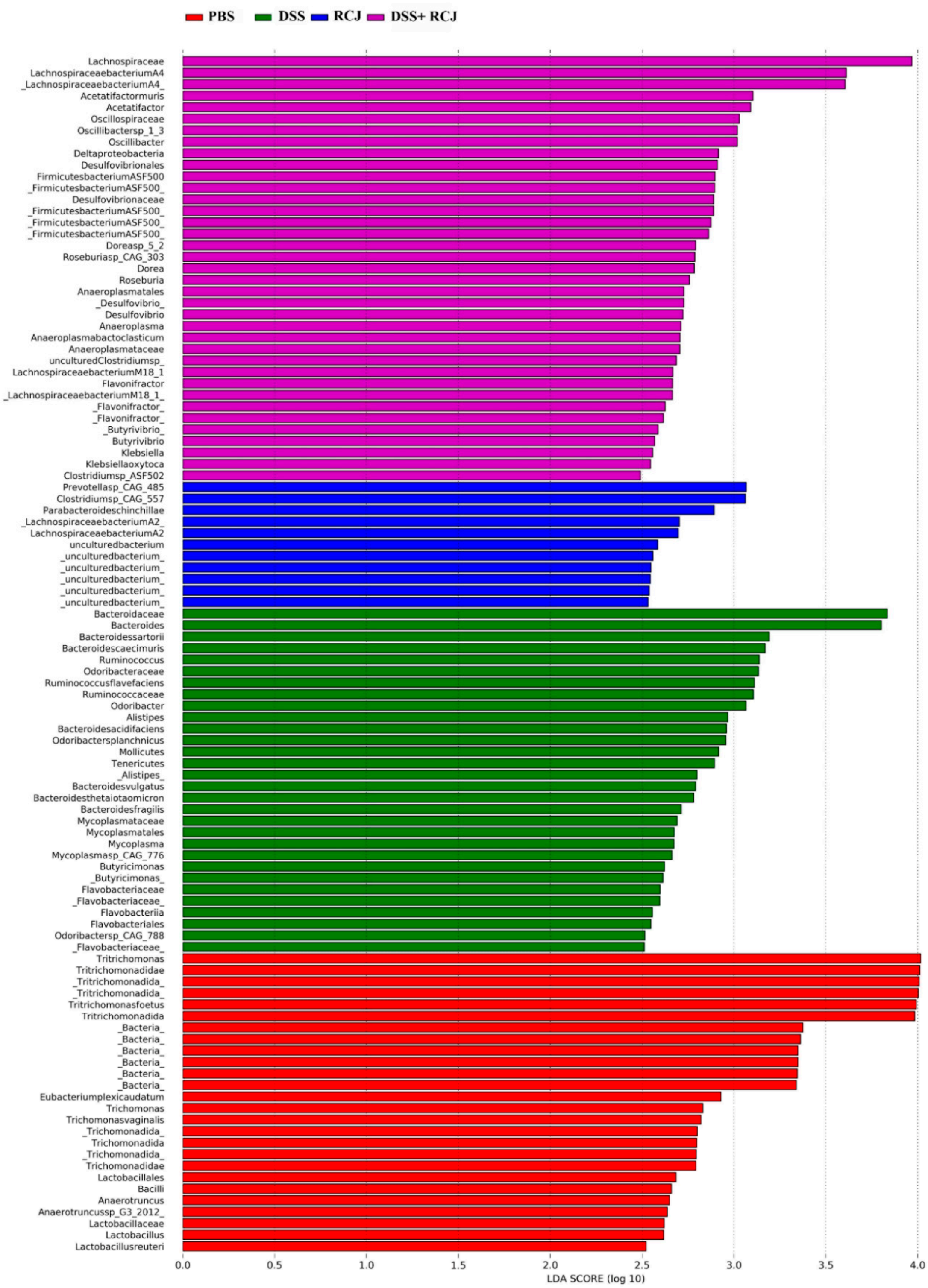
PBS
DSS
RCJ
DSS+ RCJ
Lachnospiraceae
LachnospiraceaebacteriumA4
LachnospiraceaebacteriumA4
Acetatifactormuris
Acetatifactor
Oscillospiraceae
Oscillibactersp_1_3
Oscillibacter
Deltaproteobacteria
Desulfovibrionales
FirmicutesbacteriumASF500
FirmicutesbacteriumASF500
Desulfovibrionaceae
FirmicutesbacteriumASF500
FirmicutesbacteriumASF500
FirmicutesbacteriumASF500
Doreasp_5_2
Roseburiasp_CAG_303
Dorea
Roseburia
Anaeroplasmatales
Desulfovibrio
Desulfovibrio
Anaeroplasma
Anaeroplasmabactoclasticum
Anaeroplasmataceae
unculturedClostridiumsp_
LachnospiraceaebacteriumM18_1
Flavonifractor
_LachnospiraceaebacteriumM18_1_
Flavonifractor
Flavonifractor
Butyrivibrio
Butyrivibrio
Klebsiella
Klebsiellaoxytoca
Clostridiumsp_ASF502
Prevotellasp_CAG_485
Clostridiumsp_CAG_557
Parabacteroideschinchillae
LachnospiraceaebacteriumA2
LachnospiraceaebacteriumA2
unculturedbacterium
unculturedbacterium
unculturedbacterium
unculturedbacterium
unculturedbacterium
unculturedbacterium
Bacteroidaceae
Bacteroides
Bacteroidessartorii
Bacteroidescaecimuris
Ruminococcus
Odoribacteraceae
Ruminococcusflavefaciens
Ruminococcaceae
Odoribacter
Alistipes
Bacteroidesacidifaciens
Odoribactersplanchnicus
Mollicutes
Tenericutes
Alistipes
Bacteroidesvulgatus
Bacteroidesthetaiotaomicron
Bacteroidesfragilis
Mycoplasmataceae
Mycoplasmatales
Mycoplasma
Mycoplasmasp_CAG_776
Butyricimonas
Butyricimonas
Flavobacteriaceae
Flavobacteriaceae
Flavobacteriia
Flavobacteriales
Odoribactersp_CAG_788
Flavobacteriaceae
Tritrichomonas
Tritrichomonadidae
Tritrichomonadida
Tritrichomonadida
Tritrichomonasfoetus
Tritrichomonadida
Bacteria
Bacteria
Bacteria
Bacteria
Bacteria
Bacteria
Eubacteriumplexicaudatum
Trichomonas
Trichomonasvaginalis
Trichomonadida
Trichomonadida
Trichomonadida
Trichomonadidae
Lactobacillales
Bacilli
Anaerotruncus
Anaerotruncussp_G3_2012_
Lactobacillaceae
Lactobacillus
Lactobacillusreuteri
0.0
0.5
1.0
1.5
2.0
2.5
3.0
3.5
4.0
LDA SCORE (log 10)

Figure S2. Shows **LefSe** analysis to detect significantly different taxa at the different taxonomic levels

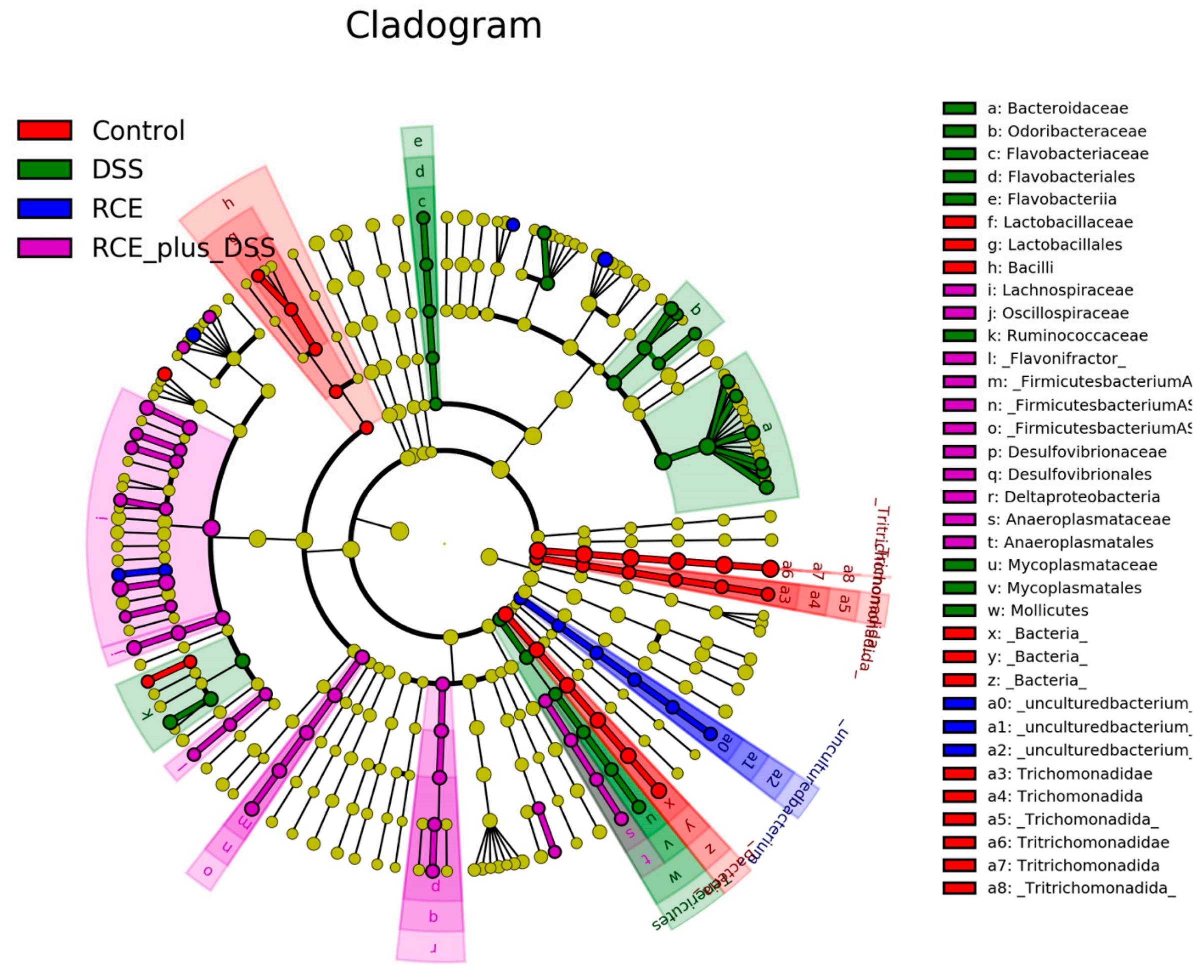

Figure S3. The cladogram showed substantial differences in 106 taxa among four treatment groups (PBS, RCJ, DSS, and DSS+RCJ) Red, green, blue, and purple indicate different groups, with the species classification at the phylum level, class, order, family, and genus shown from the inside to the outside

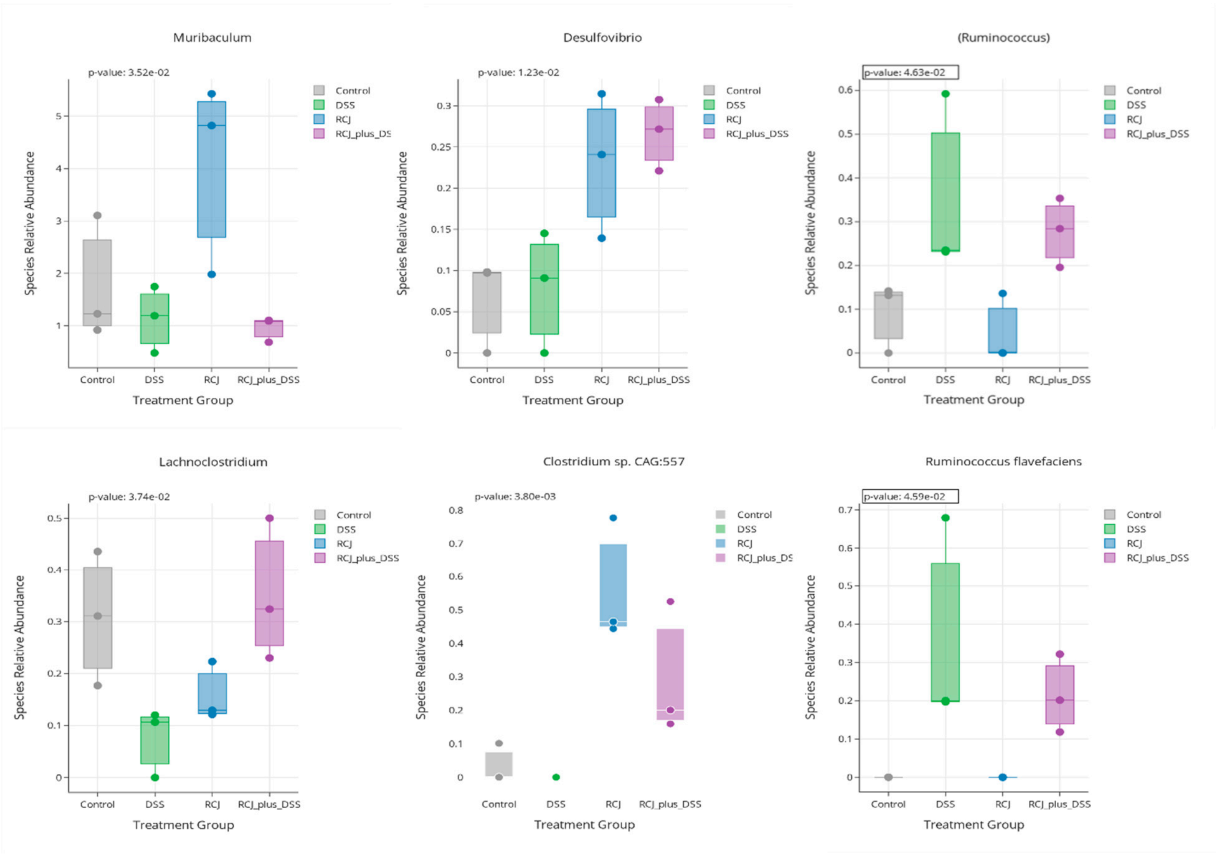

Figure S4. Graphs show the relative abundance of significant organisms at phylum, genus, and species level.

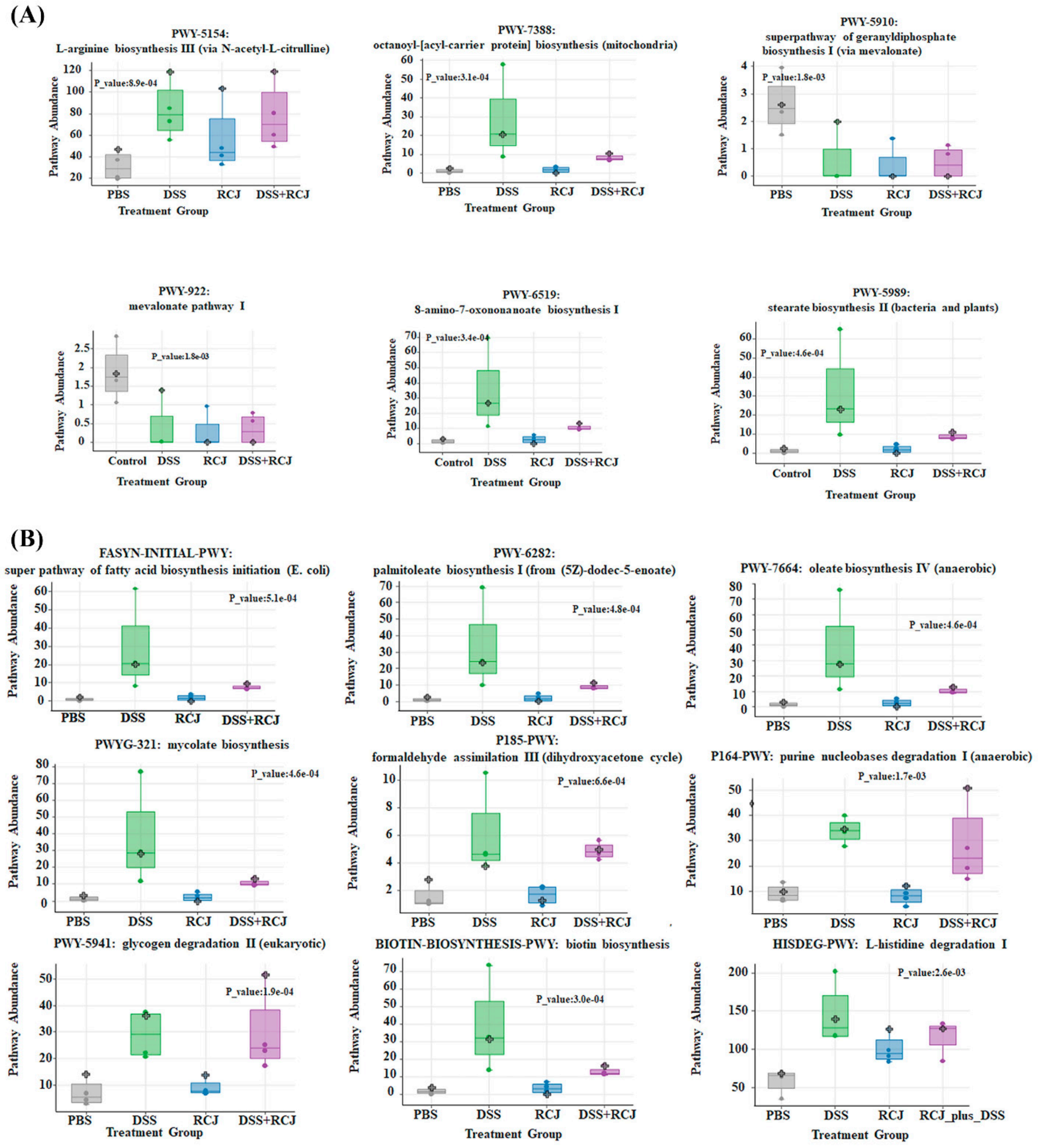

Figure S5. (**A**,**B**) Indicates the rest of the top significantly regulated pathways beneficial for colon epithelium health.
